# Possible association of Typhoon Hagibis and the COVID‐19 pandemic on patient delay in breast cancer patients: A case report

**DOI:** 10.1002/ccr3.5621

**Published:** 2022-03-22

**Authors:** Yudai Kaneda, Akihiko Ozaki, Masahiro Wada, Tomohiro Kurokawa, Toyoaki Sawano, Masaharu Tsubokura, Tetsuya Tanimoto, Yoshiaki Kanemoto, Tomozo Ejiri, Norio Kanzaki

**Affiliations:** ^1^ 12810 School of Medicine Hokkaido University Sapporo Hokkaido Japan; ^2^ 37013 Department of Breast Surgery Jyoban Hospital of Tokiwa Foundation Iwaki Fukushima Japan; ^3^ 37013 Department of Breast Surgery Sano Kosei General Hospital Sano Tochigi Japan; ^4^ 37013 Department of Surgery Jyoban Hospital of Tokiwa Foundation Iwaki Fukushima Japan; ^5^ 183174 Department of Radiation Health Management Fukushima Medical University School of Medicine Iwaki Fukushima Japan; ^6^ 37013 Department of Internal Medicine Jyoban Hospital of Tokiwa Foundation Iwaki Fukushima Japan

**Keywords:** breast cancer, COVID‐19, disasters, Japan, Typhoon Hagibis

## Abstract

Little is known on how different types of disasters interact in their impacts on patient care. We experienced a breast cancer patient whose initial presentation was delayed for 2 years due to the COVID‐19 pandemic and Typhoon Hagibis. Increasing awareness is needed on the combined impacts of disasters on breast cancer management.

## Graphical abstract

Timely presentation is critical in breast and other cancer management, but disasters may hamper these processes. Particularly, disaster impacts on cancer care can be intensified when combined, which should be noted during the COVID‐19 pandemic.

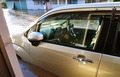

## 1 INTRODUCTION

Breast cancer is the most common cancer for women, with an estimated 2.3 million cases worldwide in 2020.^1^ Any delay in initial medical consultation and subsequent breast cancer treatment increases the risk of relapse, recurrence, and mortality.^2^, ^3^, ^4^ Therefore, it is important for the general public to be more aware of the symptoms and signs of breast cancer, and to visit medical institutions if they experience any symptoms that may lead them to suspect breast cancer.^5^, ^6^

More than 75% of breast cancer patients are detected after a recognition of their symptoms,^3^ and 20%–30% of symptomatic patients experience a delay of more than 3 months between the initial perception of symptoms and the first visit to medical institutions.^4^ From a previous study, even a three‐month delay in diagnosis has been shown to reduce the 5‐year survival rate by about 7%.^3^ For these delays, various factors related to the patients and their environment, such as young age and living alone, have been suggested.^7^

Recently, the impact of disasters on cancer care has been attracting global attention, as seen in past events such as the Triple Disaster of 2011 (earthquake, tsunami, and nuclear power plant accident), Hurricane Katrina, Gorsica earthquake, and Typhoon Hagibis. Especially with the COVID‐19 pandemic that has been spreading worldwide since November 2019, various aspects of breast cancer care have been affected negatively.^8^, ^9^ In the United States, at the peak of the pandemic in April 2020, the number of people receiving breast cancer screening decreased by 85%,^10^ and a similar decrease was reported in Japan, especially during the declaration of a state of emergency.^11^

We previously reported on how complex disasters can jeopardize cancer treatment in the context of the 2011 Triple Disaster.^12^, ^13^ However, the effects of this disaster were mainly due to the accident at the Fukushima Daiichi Nuclear Power Plant, which forced local residents to evacuate on a large scale for fear of the radiation. There is not enough information on how cancer treatment would be compromised in the event of such a combination of completely different disasters. It is clinically important to consider this issue because we are currently living under the COVID‐19 pandemic and we do not know when other disasters will occur.^14^, ^15^

Here, we report on a breast cancer patient in Fukushima, whose initial visit to medical institutions was delayed by 2 years due to Typhoon Hagibis and the COVID‐19 pandemic.

## 2 CASE PRESENTATION

A woman in her 50s living at home with her mother in her 80s needing nursing care visited our hospital in Iwaki City, Fukushima Prefecture for the first time in April 2021 due to a lump in her right breast that she had been aware since April 2019. She made her first visit to our hospital when she started to feel pain around February 2021. As a result of various examinations including mammography, ultrasonography, CT, and PET scans, she was diagnosed with Clinical T2N1M1 (bone) Stage IV breast cancer.

A detailed interview with the patient revealed her complicated situation behind the two‐year delay in initial medical consultation. This delay was due to a combination of various factors at work and at home, in addition to the 2019 Typhoon Hagibis and the COVID‐19 pandemic, which have significantly lowered the priority of receiving medical consultation. She first became aware of her symptoms in April 2019. While she considers herself to be relatively weak and not tolerant of pain, she did not have pain and could not recognize the seriousness of her condition, and used ointments to relieve them. While her symptoms did not disappear, she was not worried about it so much for a few months.

Then, she experienced the Typhoon Hagibis in October 2019. She was living near the Natsui River, which flooded during the typhoon,^16^ and the first floor of her house was completely flooded that day. She climbed up the stairs carrying her mother on her back and escaped from the flooding. A few months after the typhoon, she became aware that the lump was gradually getting larger and considered an initial consultation about her condition, as hospital fees were exempted until March 2020 due to the typhoon. However, she was living on the second floor of her house at that period and was busy cleaning the first floor, and since neither she felt any pain during the day nor knew where she could easily visit, she was reluctant to visit a hospital. Then, from around March 2020, the effects of the COVID‐19 pandemic began to appear also in Iwaki City.^16^

In April 2020, a nationwide state of emergency against the COVID‐19 pandemic was declared for the first time in Japan.^17^ Subsequently, as the pandemic had not been completely controlled in Japan, people refrained from going out as much as possible and continued to do so even after the state of emergency was ended. In addition, vaccination against the COVID‐19 was not available until the early summer of 2021. In Japan, infected people tended to be subjected to stigma,^18^ so she could not visit a hospital or even meet her relatives or friends even in this period when the state of emergency had been lifted. Moreover, the patient usually went out for work during the day and had to go home to take care of her mother as soon as she finished her work. Due to these reasons, her initial medical consultation was delayed for 2 years.

Since there was only one bone metastasis at the time of diagnosis, we discussed the advantages and disadvantages of each treatment strategy with the patient and performed chemotherapy followed by mastectomy and axillary dissection in September 2021. She is treated with the hormonal therapy with fulvestrant and abemaciclib as of October 26, 2021.

## 3 DISCUSSION

We reported a case of advanced breast cancer patient whose initial diagnosis was delayed by 2 years in Fukushima, Japan, mainly due to the combined effects of Typhoon Hagibis and the COVID‐19 pandemic. If she had been consulted earlier, she might have been able to receive treatment for her breast cancer before she developed bone metastasis.

This case shows that undiagnosed breast cancer patients tend to postpone their first medical consultation in the event of a disaster, and this effect may be strengthened especially for patients who tend to delay seeking help. A notable finding of this case is that living with other family members partly prevented her from making initial medical consultation. Although social support from family or friends is extremely important for the treatment and recovery process of breast cancer patients,^19^ it may also have a negative impact as shown in this case. Therefore, it is important to understand the characteristics of each patients and treat them individually.

At the same time, it is important to recognize that delays in medical consultation may occur before healthcare professionals and others have contact with those patients, which may limit the actual measures taken. In order to deal with this problem, it is essential to cooperate with media such as newspapers and television to further raise people's awareness of breast cancer symptoms and encourage patients to accurately recognize the severity of their symptoms so that they can seek initial advice from their family or friends. Also, especially in this case, it would have been necessary to intervene to her workplace or home to ensure that the patient could receive appropriate medical care, for example, by establishing an environment where the patient could easily take time off work, or by sending someone who could take care of her mother instead of her.

In addition, in the event of a disasters during the COVID‐19 pandemic, it is necessary to handle the situation with precautions to prevent the spread of infection. Generally, those affected by disasters are vulnerable to infectious diseases due to overcrowded shelters, environmental changes, and poor personal hygiene.^20^ Furthermore, since many people have refrained from social interactions for fear of infection, various support activities that have been carried out in the past disasters, such as child care, transportation of supplies, and soup kitchens,^21^ may be reduced. In order to ensure access to medical services even in such a situation, it is also important to share information on hospitals and evacuation centers where medical treatment is available, and to expand telemedicine services.

In conclusion, we experienced a breast cancer patient whose medical consultation was delayed for a long time due to the combined effects of the COVID‐19 pandemic and the typhoon. Since such health problems may also occur in disasters other than typhoons shown in this case, it is necessary to conduct larger cohort studies in the future. In addition, especially in the current situation of the COVID‐19 pandemic, it is important to communicate the risks of breast cancer and the benefits of screening by effectively utilizing the media to encourage patients to seek timely medical care. Moreover, it is important to approach patients individually by providing consultation services for patients and their families, or reminding patients to visit the hospital.

## CONFLICT OF INTEREST

Akihiko Ozaki receives personal fees from MNES Inc., outside the submitted work. Also, Tetsuya Tanimoto receives personal fees from MNES Inc., and Bionics co., ltd. outside the submitted work. No other authors reported conflicts of interest.

## AUTHOR CONTRIBUTIONS

Kaneda Y and Ozaki A involved in conception and designing of the study. Kaneda Y wrote this paper. Ozaki A involved in critical revision of the paper. All the authors read the final draft and approved submission.

## ETHICAL APPROVAL

None.

## CONSENT

Written informed consent was obtained from the patient to publish this report in accordance with the journal's patient consent policy.

## Data Availability

None.
